# Three Cases of Intestinal Obstruction

**Published:** 1884-09

**Authors:** Alfred Sheen

**Affiliations:** Senior Surgeon, Cardiff Infirmary


### Clinical Records.

---

### THREE CASES OF INTESTINAL OBSTRUCTION.

By ALFRED SHEEN, M.D., Senior Surgeon, Cardiff Infirmary.

*Case* I.—James Searle, a sailor, age 20, single, was admitted to the Cardiff Infirmary on March 26th last, at noon, with the following history, the notes being taken at 3 p.m.:—

On the 16th, *i.e.*, ten days before his admission, he had what he called a "stoppage of water." He took an aperient pill on the 17th, which acted well on the 18th. He was frequently sick on the 17th, 18th, and 19th, but afterwards began to get better. The bowels were moved on the 19th. He then got about and was "all right," he having a daily motion till the 24th. On that day he was obliged to go to bed, suffering from pain in the abdomen, so severe that he could scarcely bear the weight of the bed-clothes. He was constantly sick all that afternoon, bringing up brownish fluid, which had a very disagreeable smell. The bowels were relieved two or three times on the 23rd, and once on the 24th, but not since, neither has he passed any fæces. He had medicine from a doctor yesterday and to-day. Such is the history of the case as given by the patient.

He has been constantly sick since admission, bringing up a brown fluid with a fæcal smell. Countenance anxious, eyes sunk, cheeks sunk in, extremities cold, pulse small, quick; pain in abdomen round navel, but not so bad as it has been; tongue clean, moist; T. 97°; no abdominal distension; tender surface, dull on percussion, to the left and below the umbilicus; no blood or pus passed by the bowels.

Soap and water was at once injected freely into the bowels, which brought away a little fæces. The symptoms continued. As he refused operative treatment, he was ordered small quantities of ice and brandy, and liq. op. Sed. m. v. T. Belladon. m. x. every half hour. By-and-by he consented to allow me to do what was thought best, and at 7 p.m., after a consultation, I opened the abdomen by an incision along the linea alba, below the umbilicus, and seizing the first piece of intestine which presented at the wound, and which was very red, stitched it to the abdominal wall and then opened it. There was a gush of milky-looking fluid, a small quantity of which escaped into the abdomen. There was no sickness after the operation, but the collapse continued. The patient died at 4 a.m. on the 27th, eleven hours after the operation, a great deal of fluid escaping through the opening during the night.

*Necropsy thirty hours after death.* (By Mr. Griffiths, M.B.Lond., House Surgeon.)—Edges of wound pretty firmly united to abdominal wall. On opening the abdomen, the intestines were found to be of a brightish red colour, smooth surface, covered with lymph, with small quantities of intestinal fluid between the coils, which in some places were adherent. The large omentum, almost devoid of fat, and of a red colour, was firmly fixed to the pelvic wall; on separating this, a large coil of intestine was found firmly bound down by the omentum into the cavity of the pelvis.

The large intestine was empty and contracted.

*Case* II.—Jane Edwards, æt. 33, married, was admitted into the Cardiff Infirmary on the morning of April 14th, 1884, looking seriously ill. She had been under the care of Dr. Fiddian, who said she had had a miscarriage six months previously, and since then she had not been in her usual health. About fourteen days ago he was called to see her, and found her suffering from pain in the right side of the abdomen, and at the same time he observed a tumour in the right lumbar region. He gave her medicine, which relieved her pain. A few days ago he was called up in the night, the patient suffering severe pain in the same situation, which was relieved by an hypodermic injection of morphia. She says her bowels were relieved three days ago, since which there has been no motion, although her husband says there has, and sickness has been constant.

On admission she had a pinched look, and was suffering from collapse; p. 128, T. 98°; sick after everything she takes. The abdomen was not swollen. On the right side, towards the lumbar region, there was increased resistance, and a distinct swelling to be felt, although it could not be seen. No blood has been passed by the bowels. The immediate symptoms were those of intestinal obstruction. A consultation was called, and the diagnosis formed was that there was considerable thickening, probably from inflammatory mischief, at the seat of pain, and that the bowels had become involved in this; and it was decided that opening the abdomen did not offer her a fair chance of relief. She died on the evening of the 15th. It was thought probable by one or two who saw the patient that the tumour might be connected with the kidney; the short duration of the symptoms seemed against this supposition, which, however, at the *post mortem,* turned out to be correct.

On opening the abdomen after death, a very rare condition of things was found. A smooth, oval-shaped tumour was seen lying in the right lumbar region, the upper part of which was black, and was lying on some coils of the upper portion of the small intestines. The intestines from this point onwards, towards the rectum, were flat, contracted, and empty, but otherwise normal in appearance; and on lifting the tumour they immediately and slowly filled with air from the stomach and intestine above. On tracing the tumour, which was about the size of an elongated cocoanut, it was found to be a mass of encysted, coagulated blood, surrounding the kidney; and this blood was traced to a small anuerism (?) about the size of a pea, situated in the substance of the kidney, close to its outer surface. There are two points of interest about this case:—1. The rarity of the disease. 2. The facility with which the intestines became filled with air after lifting the tumour. This latter made me regret that I had not opened the abdomen; but it is easy to be wise after the event.

Mr. Savage says, in the *British Medical Journal,* Vol. i. 84, 454, speaking of abdominal section in these cases:—"In many instances where the case requires surgical aid, it is both useless and mischievous to make a long incision and insert the whole hand in the abdomen, amongst a mass of distended intestine, in search of the cause of obstruction, the relief of which is not always practicable. Recoveries after such procedures are but few. It is better practice to make a small opening, seize the first part of distended intestine that presents itself, stitch it to the wound, including the peritoneum, and then open it and give free exit to retained fæces." This operation was performed in the first case, and, as an operation, was successful; but the patient was too far gone to recover; he never rallied from his previous state of collapse. An interesting point at the *post mortem* was the fact that only twelve hours after the operation the parietal and intestinal surfaces of peritoneum were pretty firmly united.

*Case* III.—A male child, born on the evening of June 7th, 1884. I saw the child, in consultation with Mr. W. D. J. Morris, on the 10th. There had been some retention of urine, owing, probably, to blocking of the urethra, as this symptom disappeared on some muco-pus passing from the urethra. Nothing had been passed per anum. On examination the anus was found perfect, contracting on the finger; but the finger could not be introduced beyond two inches, where the rectum terminated in a cul de sac; there was no bulging of the parts beyond when the child cried. A trochar and camula was passed into the rectum through the upper closed extremity. This was at 5 p.m. The case did not seem favourable for further operative interference. There was much tympanites, and this was relieved by puncture with Southey's trochar and canula. The child died at midnight.

A *necropsy* on the 11th revealed some fæcal extravasation into the abdominal cavity from thinning and giving way of the intestine. (This was not at the seat of puncture.) The large intestine terminated in a cul de sac adherent to the pelvic wall, and close to the distal part of the rectum reached from the anus.

Here are three interesting cases of intestinal obstruction, each differing widely as to its cause, and all ending in death. Could a different result have been looked for under any conditions of treatment? I think not. In our treatment of these lesions one great obstacle to possible success seems to me to be the difficulty of making a differential diagnosis. Mr. H. O. Thomas, in his work on *Intestinal Obstruction*, says:—"This is not of vital importance. It would be valuable; but as it is difficult, it is satisfactory to know that curative treatment can be practised without it." A second obstacle in many cases is the active medicinal and mechanical treatment the patients have previously undergone. I think there can be no doubt that purgatives, enemata, introduction of a long tube, sweating, and inversion, &c., so far from doing good, are calculated to do much harm, besides, perhaps, entailing the loss of much valuable time. So far as my reading and experience goes, all such remedies make the patient worse, and lessen his chances of recovery, where recovery might otherwise be possible. (The use of an enema in Case I. was obviously bad treatment.) What we want to know more clearly than we do now is, what are the cases in which we may fairly rely upon treatment by sedatives, rest, and abstention from food, and what are the cases where abdominal section should be performed? Thomas, in the work referred to, gives us some guides, but they do not appear to me to be sufficient. He says:—

"It cannot be judicious surgery to perform haphazard operations because in some isolated cases the *post mortem* shews that an operation *might* have saved the patient."

"No operation should be performed unless the surgeon is convinced that it is necessary." Query.

"An *early* operation *may* be attended with better success, but only because by it the patient escapes a long period of higgledy-piggledy treatment, still in vogue. This is all that can be advanced at the present time in favour of early direct interference by abdominal section." Query.

"Is an operation required? Consider, first, the duration of the disease; second, the present condition, or that which it shews to be imminent."

"1. *Duration of the disease.*—If the patient's primary treatment has been proper, it is highly improbable that an operation will be required earlier than the third day, or later than the third week. In most instances the period for operating will be indicated about the seventh day." In what way?

"2. *Condition of the patient.*—If the pulse early continue rapid, or at a late period become so, its rate being only a little reduced or its volume increased by the opiate, with typhoid tongue, intense thirst, vomiting more than four times in the twenty-four hours, with or without collapse, think about operating, with hope of relief, if the preliminary treatment has not been faulty."

Dr. Clifford Allbutt states most distinctly that, in intestinal obstruction, therapeutics must depend on diagnosis; that if we will carefully attend to the case, diagnosis, as a rule, is not so very difficult; that in acute cases inflammation usually counts for a good deal, and that an important part of the treatment is to treat the inflammation by rest and opium; that in twists of the bowels, snaring in bands or fissures, knots in its length, are internal hernias, and are to be treated as such; *i.e.*, if not relieved by rest and sedatives, then by operation.

If we can diagnose these latter cases, I am clearly of opinion that abdominal section should be performed, and I am doubtful of the value of much preliminary treatment by rest and sedatives. How frequently do we witness "too late" operations, with resulting death, in cases of external hernia! and are not the conditions the same in internal hernia, and, therefore, the necessity for early operation equally strong? If, in a case of internal hernia, we open the abdomen and relieve the constriction, and the patient dies, is it not just possible that he dies because we have left him too long a time without relief? Why should we be able, even in apparently unpromising cases, to perform ovariotomy successfully, and yet not be able to treat internal hernia by abdominal section successfully?

